# Robotic‐Assisted Transbronchial Biopsy of Aortopulmonary Window Lymph Nodes Using Cone‐Beam CT Guidance

**DOI:** 10.1002/rcr2.70495

**Published:** 2026-01-29

**Authors:** Venkatkiran Kanchustambham

**Affiliations:** ^1^ Pulmonary & Critical Care Medicine Sanford Health and University of North Dakota School of Medicine & Health Sciences Fargo North Dakota USA

**Keywords:** aortopulmonary window, cone‐beam CT, mediastinal lymph nodes, robotic bronchoscopy, small cell lung cancer

## Abstract

Sampling aortopulmonary window lymph nodes is technically challenging and often requires surgical approaches. We report a minimally invasive robotic‐assisted transbronchial biopsy of AP window lymph nodes using cone‐beam CT guidance, enabling diagnosis of small cell lung cancer without surgical mediastinal biopsy.

## Graphical abstract

Robotic bronchoscopy combined with cone‐beam CT enabled minimally invasive biopsy of aortopulmonary window lymph nodes, leading to diagnosis of small cell lung cancer without surgical mediastinoscopy.

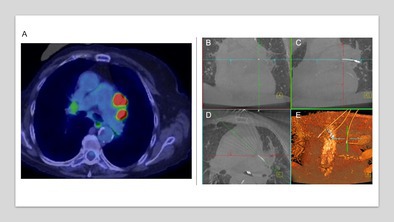

A 74‐year‐old woman with a 50 pack‐year smoking history and remote right‐sided breast cancer presented with progressive mediastinal lymphadenopathy detected on lung cancer screening surveillance. Computed tomography demonstrated interval enlargement of a left aortopulmonary (AP) window lymph node measuring approximately 2.1 cm in short‐axis diameter and extending approximately 3.6 cm craniocaudally. The lesion was located approximately 1.5–2.0 cm lateral to the distal trachea and immediately anterior to the left main pulmonary artery, abutting the aortic arch. FDG‐PET revealed intensely hypermetabolic uptake within this nodal conglomerate (SUVmax 15.4), without an FDG‐avid pulmonary mass (Figure 1A).

**FIGURE 1:**
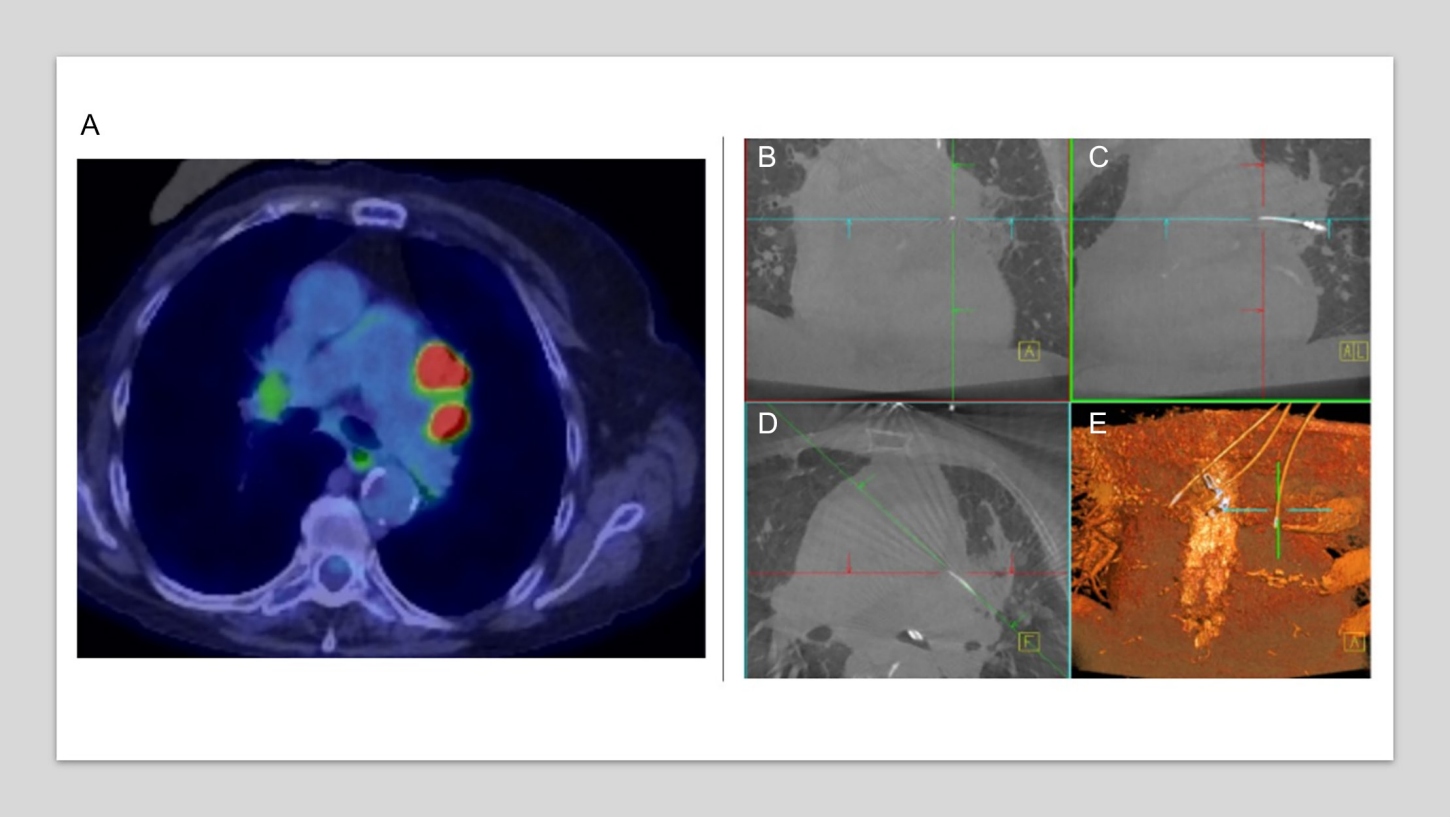
Robotic‐assisted transbronchial biopsy of aortopulmonary window lymph nodes using cone‐beam CT guidance. (A) FDG‐PET/CT demonstrating intensely hypermetabolic mediastinal lymphadenopathy involving the aortopulmonary (AP) window and left anterior hilar region, without an FDG‐avid pulmonary mass. (B, C) Cone‐beam CT axial and coronal multiplanar reconstructions confirming precise robotic catheter and biopsy needle trajectory towards the AP window lymph node adjacent to the aorta and pulmonary trunk. (D) Oblique cone‐beam CT view demonstrating needle advancement into the targeted lymph node. (E) Three‐dimensional cone‐beam CT reconstruction illustrating spatial orientation of the robotic catheter and biopsy needle, confirming safe transbronchial access to station 5 lymph nodes.

Given the nodal location adjacent to the aorta and pulmonary trunk—traditionally inaccessible by conventional bronchoscopy and often requiring surgical approaches—robotic‐assisted bronchoscopy integrated with cone‐beam CT was performed. Cone‐beam CT multiplanar and three‐dimensional reconstructions confirmed precise transbronchial catheter and biopsy needle positioning within the AP window lymph node (station 5), allowing safe tissue acquisition (Figure 1B–E). Cytology demonstrated small cell carcinoma with tumour cells positive for TTF‐1, synaptophysin, chromogranin and pan‐cytokeratin, with a Ki‐67 proliferation index > 95%, consistent with small cell lung cancer. Sampling of additional mediastinal stations (11R, 4R, 7 and 4L) was negative.

This case highlights the feasibility of minimally invasive robotic‐assisted transbronchial biopsy of AP window lymph nodes, potentially obviating the need for surgical mediastinal biopsy in selected patients [1, 2, 3].

## Author Contributions

Venkatkiran Kanchustambham was responsible for patient care, procedural planning, image selection, literature review and manuscript preparation.

## Funding

The author has nothing to report.

## Consent

Written informed consent was obtained for publication of this clinical image and accompanying material in accordance with journal requirements.

## Conflicts of Interest

The author declares no conflicts of interest.

## Data Availability

The data that support the findings of this study are available from the corresponding author upon reasonable request.
